# Ovarian fluid allows directional cryptic female choice despite external fertilization

**DOI:** 10.1038/ncomms12452

**Published:** 2016-08-16

**Authors:** Suzanne H. Alonzo, Kelly A. Stiver, Susan E. Marsh-Rollo

**Affiliations:** 1 Department of Ecology and Evolutionary Biology, Yale University, 165 Prospect Street, New Haven, Connecticut 06520, USA; 2 Department of Ecology and Evolutionary Biology, University of California Santa Cruz, 1156 High Street, Santa Cruz, California 95064, USA; 3 Psychology Department, Southern Connecticut State University, 501 Crescent Street, New Haven, Connecticut 06515, USA; 4 Department of Psychology, Neuroscience, and Behaviour, McMaster University, 1280 Main Street W, Hamilton, Ontario, Canada L8S4K1

## Abstract

In species with internal fertilization, females can favour certain males over others, not only before mating but also within the female's reproductive tract after mating. Here, we ask whether such directional post-mating (that is, cryptic) female mate choice can also occur in species with external fertilization. Using an *in vitro* sperm competition experiment, we demonstrate that female ovarian fluid (ovarian fluid) changes the outcome of sperm competition by decreasing the importance of sperm number thereby increasing the relative importance of sperm velocity. We further show that ovarian fluid does not differentially affect sperm from alternative male phenotypes, but generally enhances sperm velocity, motility, straightness and chemoattraction. Under natural conditions, female ovarian fluid likely increases the paternity of the preferred parental male phenotype, as these males release fewer but faster sperm. These results imply females have greater control over fertilization and potential to exert selection on males in species with external fertilization than previously thought possible.

## Abstract (web summary)

In some species with internal fertilization, females can mate with multiple males and then manipulate which sperm fertilize the eggs. Here, Alonzo *et al.* find that by releasing ovarian fluid along with their eggs, female ocellated wrasse are able to influence paternity despite having external fertilization.

## Graphical abstract

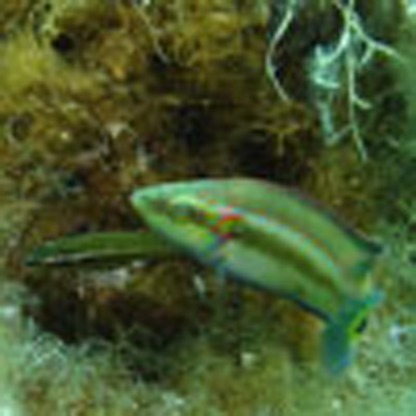

Female mate choice is a powerful force, capable of driving the evolution of male reproductive traits^1^. In species with internal fertilization, females are known to choose not only among potential mates before mating (that is, pre-mating female mate choice), but also among potential fathers after mating (that is, post-mating or cryptic female mate choice; defined here as a female-driven bias in fertilization that occurs after mating or the release of gametes)^2^,^3^,^4^,^5^,^6^. It is often assumed, however, that females in species with external fertilization are limited to pre-mating choice because a female cannot influence fertilization when it happens outside of her body. Paradoxically, it is precisely in these species—where females have limited control over which males compete to fertilize their eggs—in which selection is particularly expected to favour cryptic (or post-mating) female choice^7^. The open question is whether mechanisms exist that allow females to bias the outcome of sperm competition among conspecific males toward a consistently preferred adult male phenotype even when fertilization is external.

In recent years, mechanisms at the gametic level have been identified that reduce hybridization and polyspermy in species with external fertilization^8^,^9^,^10^,^11^,^12^,^13^,^14^. Recent evidence also suggests that compounds such as ovarian fluid (ovarian fluid, produced by females and released with the eggs) and gamete-recognition proteins can bias fertilization toward genetically compatible conspecific males in species with external fertilization^7^,^13^,^15^,^16^,^17^,^18^,^19^,^20^,^21^. In general, female ovarian fluid has been found to increase sperm velocity, motility, longevity and chemoattraction^18^,^22^,^23^,^24^,^25^,^26^,^27^,^28^,^29^,^30^,^31^. Ovarian fluid has also been shown to mediate sexual selection in one species with internal fertilization^6^, suggesting that it could also affect sexual selection in species with external fertilization^7^. However, none of the mechanisms identified to date allow directional cryptic female choice in a species with external fertilization and, therefore, also do not generate directional sexual selection on males within a species^3^,^7^,^32^.

Here, we ask whether female ovarian fluid provides a mechanism by which females can exhibit directional cryptic female mate choice—thus exerting directional sexual selection on adult male traits—even when fertilization happens outside of her body. To address this question, we performed a series of experiments that examine how female ovarian fluid affects the outcome of sperm competition and sperm characteristics in a species of fish with external fertilization and intense sperm competition. In the ocellated wrasse (*Symphodus ocellatus)*, three distinct alternative male types occur^33^,^34^,^35^. Large colourful parental (nesting) males build nests of algae, court females and care for eggs. Smaller non-parental (sneaker) males join a nesting male and a female during mating, but do not court females or provide care. Satellite males are intermediate in size and colour, court females, chase away sneaker males, join the nesting male and female when they spawn, but do not help with parental care^34^. Sneakers and nesting males represent separate life history pathways determined by early differences in growth^35^: a slow-growing juvenile male will be a sneaker in his first breeding season and a satellite male in his second; a fast-growing male will be a satellite in his first breeding season and a nesting male in his second. While it is clear that these alternative phenotypes are not the result of a simple genetic polymorphism, the degree to which differences in early growth are environmentally versus genetically determined is not known. Our experiments therefore focused on sperm competition between males on separate life history pathways (that is, sneakers and nesting males), as they are known to differ in physiology, behaviour, sperm allocation, age and early growth^34^,^35^,^36^,^37^,^38^,^39^,^40^, and females exhibit a strong pre-mating preference for nesting males^41^. Independent of the degree to which these alternative phenotypes are genetic or condition-dependent in origin, female mate choice can generate directional sexual selection on males that affects their evolution and expression^41^,^37^. Here, we consider if and how directional cryptic female mate choice affects selection on these alternative male phenotypes.

As in most species with external fertilization, a female ocellated wrasse can control when and where she releases her eggs, but not which males contribute sperm that compete to fertilize her eggs. Spawning is demersal: females release a batch of eggs that stick to the algae of the nest and males release sperm in quick succession and close proximity to the eggs^33^,^34^. Females of this species consistently exhibit a strong pre-mating preference for nesting males^41^,^42^. Nesting males are older, grow more quickly as juveniles, provide parental care and have higher reproductive success than sneaker males^35^. If nesting males sire offspring that are more likely to grow quickly, survive and (if male) become nesting males, a general (or consensus) pre-mating female preference for nesting males would be adaptive. Yet, females cannot avoid sneaker males because fertilization is external^38^. Selection would therefore likely also favour, if available, a female-driven mechanism that allows directional post-mating female choice that favours the paternity of the nesting male phenotype in this species. Such directional cryptic female choice would exert sexual selection on these alternative male phenotypes, potentially influencing their evolution and expression.

Using a series of experiments on the ocellated wrasse, we examined whether ovarian fluid provides such a mechanism of directional cryptic female mate choice and asked how ovarian fluid affects male sperm characteristics and chemoattraction. *In vitro* sperm competition trials were used to isolate post-mating female choice (driven by ovarian fluid) from other factors affecting sperm competition and fertilization. The ovarian fluid was either left untouched, removed, or removed and replaced. Sperm from a sneaker and a nesting male were then simultaneously added to the eggs, videotaped to determine sperm number and characteristics, and parentage assigned. These data allowed us to ask how the presence or absence of ovarian fluid affected paternity and the relationship between paternity and sperm number. We performed two additional experiments to determine the effect of ovarian fluid on sneaker and nesting male sperm characteristics and chemoattraction.

These experiments show that female ovarian fluid changes the outcome of sperm competition by decreasing the importance of sperm number, thereby increasing the relative importance of sperm velocity. We therefore demonstrate that female ovarian fluid provides a mechanism by which females can bias the outcome of fertilization toward certain male phenotypes, even in species with external fertilization. Such directional cryptic female mate choice can exert post-mating sexual selection on male sperm characteristics and reproductive allocation, and thus shape the evolution of male phenotypes. We further show that ovarian fluid does not differentially affect sperm from alternative male phenotypes, but generally enhances sperm velocity, motility, straightness and chemoattraction. Under natural conditions, female ovarian fluid likely increases the paternity of the preferred parental male phenotype, as nesting males release fewer but faster sperm than sneaker males. ovarian fluid may therefore also allow females to exert directional cryptic female choice among male alternative reproductive types and increase the paternity of nesting males by decreasing the numbers advantage sneaker males would otherwise have during sperm competition.

## Results

### The effect of ovarian fluid on the outcome of sperm competition

**Figure 1:**
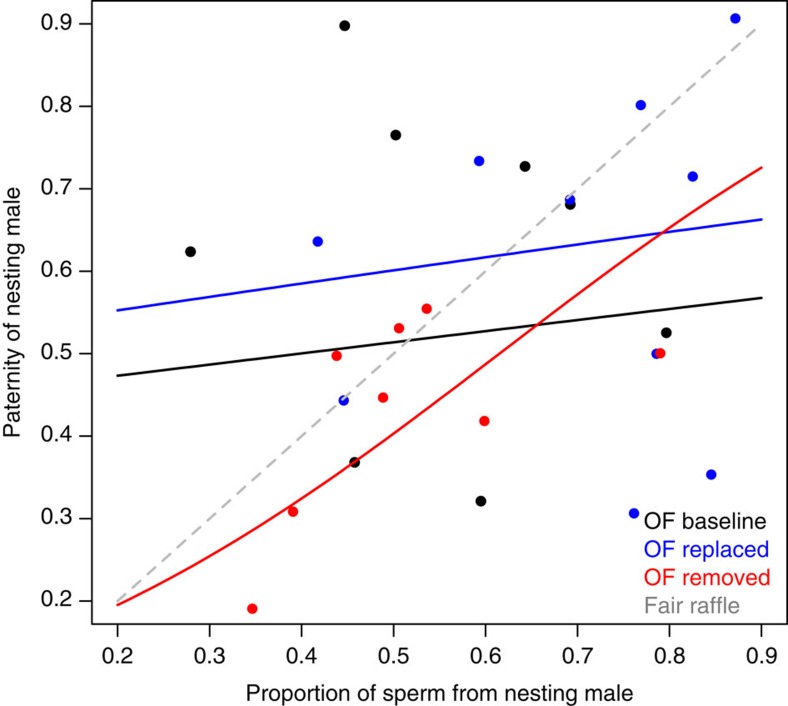
Female ovarian fluid reduces the importance of sperm number. Sperm competition changes when ovarian fluid is present to favour the paternity of the male with low sperm numbers relative to their expected paternity in the absence of ovarian fluid. Filled circles represent observed paternity (proportion of offspring sired) of the nesting male (black=ovarian fluid baseline, blue=ovarian fluid replaced and red=ovarian fluid removed). Solid black, blue and red lines show the results of the general linear logistic regression model of the form Pr (nesting male paternity, sneaker male paternity)=Relative sperm number (NM:SN) × Treatment (assuming a quasibinomial distribution of the errors due to overdispersion of the data, *n*=30, 10 per treatment)^56^, while the dashed grey line shows the expected relationship under the fair raffle model^43^,^57^. See Table 1 for full details of the best-fit model and the ‘Methods' section for further details.

**Figure 2:**
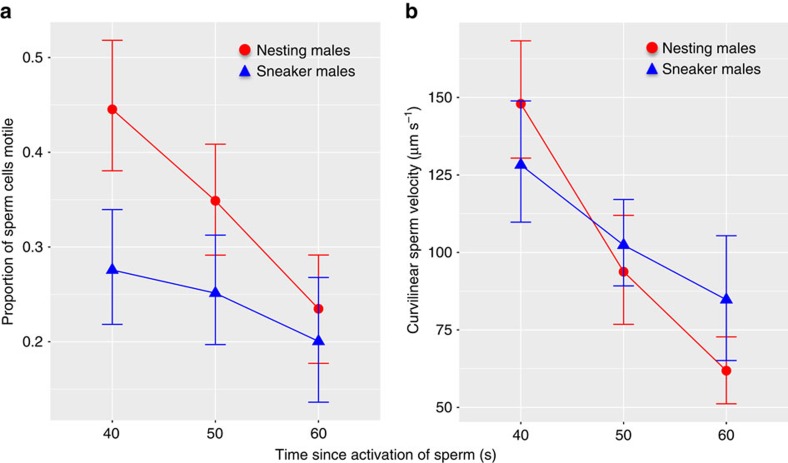
Sperm characteristics differ between nesting males and sneakers. Nesting male sperm are represented by red circles, sneaker male sperm by blue triangles. (**a**) Nesting male sperm exhibits significantly higher motility (per cent of sperm cells motile) than sneaker male sperm, and motility drops—as expected—for both male types over time. (**b**) Nesting males have higher curvilinear sperm velocity (VCL) than sneaker males shortly after activation (when fertilization is most likely to occur), but nesting male sperm velocity drops more quickly over time than sneaker male sperm velocity. Error bars indicate the s.e.m. The same qualitative pattern holds for other measures of sperm velocity (that is, straight line velocity (VSL) and average path velocity (VAP)). Statistical results are given in Table 2 (*n*=30, 10 per treatment).

In the absence of a fertilization bias, relative sperm number should determine relative paternity (for example, if sperm competition between nesting and sneaker males is simply a numbers game otherwise known as a fair raffle^43^). A male-driven fertilization bias (for example, if one male type produces sperm more able to fertilize the eggs) would be indicated by a significant effect of male type either on paternity or on the relationship between relative sperm number and paternity. In contrast, a significant effect of ovarian fluid treatment on paternity or a significant interaction between treatment and relative sperm number indicates a female effect (through the ovarian fluid) on the outcome of sperm competition among males. In this experiment, variation in paternity was best explained by variation in relative sperm number, relative sperm velocity and the interaction between ovarian fluid treatment and relative sperm number (Fig. 1, Table 1). There was, however, no main effect of ovarian fluid treatment on nesting male paternity (Table 1). We therefore find no evidence that ovarian fluid directly favours one male type (for example, nesting males) over the other (for example, sneaker males). Instead, the effect of sperm number on paternity was significantly different in the absence of ovarian fluid (ovarian fluid rinsed) than in either treatment with ovarian fluid present (ovarian fluid baseline and ovarian fluid replaced; Fig. 1, Table 1). The presence of ovarian fluid therefore reduced the importance of sperm number on fertilization success. Relative sperm velocity also had a significant and positive effect on paternity (Table 1), and nesting males had higher motility and initial sperm velocity than sneaker males (Table 2, Fig. 2).

### The effect of ovarian fluid on sperm characteristics

**Figure 3:**
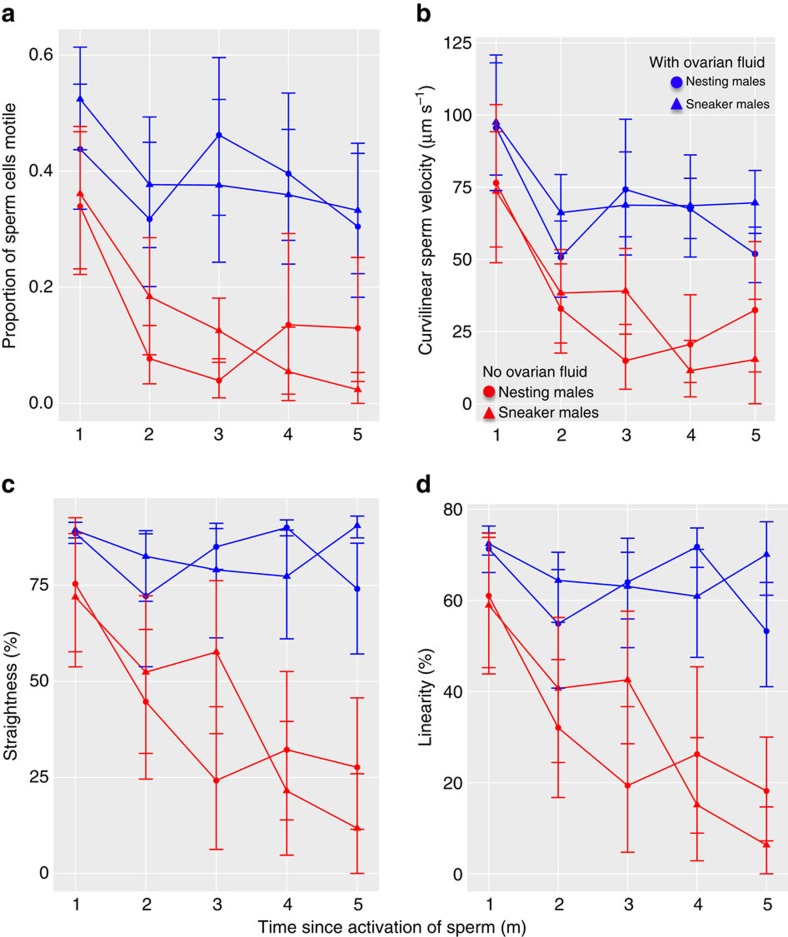
Ovarian fluid enhances both nesting and sneaker male sperm characteristics. Means for each time point and ovarian fluid treatment are represented for nesting male by circles and for sneaker males by triangles. The presence (blue) or absence (red) of ovarian fluid is represented by the colour of the symbol and lines. Error bars indicate the s.e.m. For all sperm characteristics examined except motility, the best-fit model (based on the hierarchical model and AIC comparison using ANOVA and AICctab from library bbmle in R) included treatment (with or without ovarian fluid), time (repeat) and the interaction between treatment and time (number of observations: *n*=32 males × two treatments × 5 observation points per male and treatment=320 data points). No significant effect of male type or interaction between male type and ovarian fluid treatment was found. For motility, the interaction between time and treatment was not significant. Statistical results of the linear repeated measures mixed effects best-fit models are summarized for each sperm characteristic (fit by maximum likelihood using lme from library nlme in R): (**a**) Motility (per cent of cells motile): (intercept F(158)=177.15 *P*<0.0001, treatment F(158)=106.33 *P*<0.0001, repeat F(126)=28.04 *P*<0.0001, interaction F(1)=3.02 *P*=0.084). (**b**) Curvilinear velocity (VCL): (intercept F(158)=312.21 *P*<0.0001, treatment F(158)=80.58 *P*<0.0001, repeat F(126)=42.62 *P*<0.0001, interaction F(1)=4.57 *P*=0.034). (**c**) Straightness (straight line velocity VSL/average path velocity VAP): (intercept F(158)=609.31 *P*<0.0001, treatment F(158)=147.77 *P*<0.0001, repeat F(126)=32.95 *P*<0.0001, interaction F(1)=26.54 *P*<0.0001). (**d**) Linearity (straight line velocity VSL/curvilinear velocity VCL):(intercept F(158)=545.97 *P*<0.0001, treatment F(158)=141.02 *P*<0.0001, repeat F(126)=41.51 *P*<0.0001, interaction F(1)=25.24 *P*<0.0001). Other measures of velocity (VSL, VAP) yield similar results.

**Figure 4:**
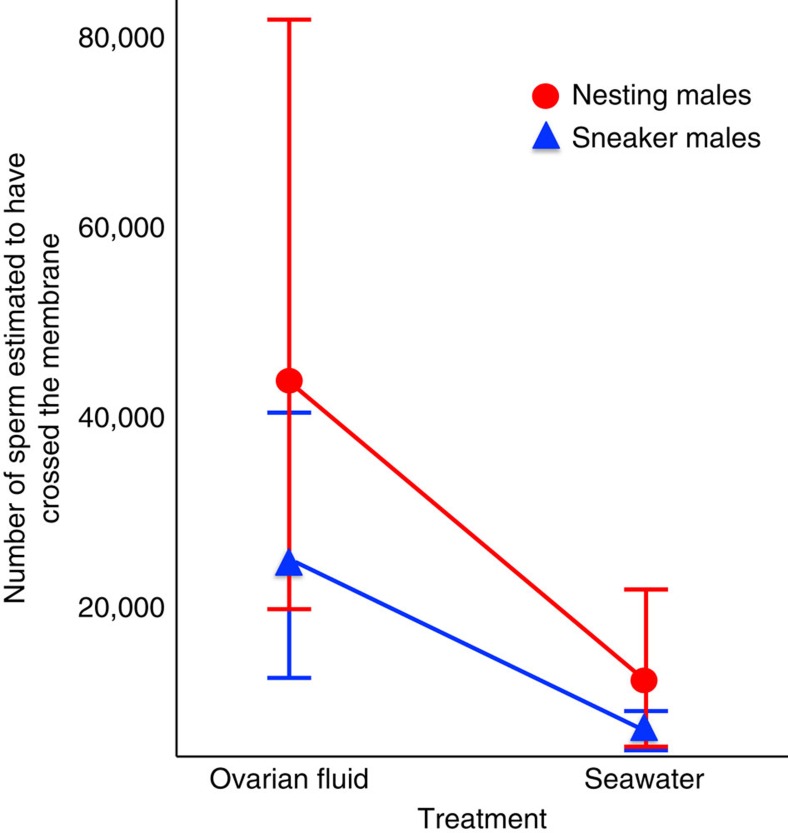
Ovarian fluid facilitates sperm chemoattraction by sneaker and nesting male sperm. Nesting male sperm are represented by red circles, sneaker male sperm by blue triangles. More sperm pass across the membrane (moving through the micropyle-sized pores) toward ovarian fluid than seawater alone. Error bars indicate the s.e.m. There is no significant effect of male type on the number of sperm that cross the membrane (*n*=60, F(1,55)=1.798, *P*=0.185, two-way ANOVA model sperm number=male type × ovarian fluid treatment, using model aov in R). There was, however, a significant effect of treatment (ovarian fluid and eggs in seawater versus seawater only) on the number of sperm found below the membrane (F(1,55)=6.758 *P*=0.012). The interaction between male type and ovarian fluid treatment was not significant (F(1,55)=0.574, *P*=0.452). Male type also did not have a significant effect on the probability that a sperm crosses the membrane (relative to the total number in the original sample, based on a general linear logistic regression model: Pr (the number of sperm above versus below the membrane)=male type+ovarian fluid treatment; *t*=−1.033, *P*=0.3061), while the presence of ovarian fluid treatment significantly increased the probability that a sperm would cross the membrane (*t*=−2.280, *P*=0.026, assuming a quasibinomial distribution of the errors due to overdispersion of the data, using glm with family=quasibinomial in R).

Having identified that female ovarian fluid provides a mechanism by which females can influence male fertilization success, we performed two additional experiments to determine how ovarian fluid affects nesting and sneaker male sperm characteristics. Using a paired design, we first compared the sperm characteristics from either a nesting or sneaker male sperm in both the presence and absence of ovarian fluid. While ovarian fluid enhances sperm velocity, motility, linearity and straightness in general, we did not detect a differential effect of ovarian fluid on sneaker versus nesting male sperm (Fig. 3). Next, we used a cell migration assay to ask whether ovarian fluid affects sperm chemoattraction. This assay examines chemoattraction of sperm toward the egg along an ovarian fluid gradient by measuring the number of sperm that swim across a membrane embedded with pores that mimic the egg's micropyle^8^ (sperm must enter fish eggs via the micropyle to initiate fertilization^44^,^45^). While ovarian fluid increases sperm chemoattraction in general (Fig. 4), we again found no evidence that ovarian fluid differentially affects nesting male sperm relative to sneaker male sperm. Thus, while ovarian fluid enhances the capacity of sperm to make it to the egg (by increasing velocity, motility, longevity, straightness and chemoattraction), it does not seem to enhance nesting male sperm characteristics *per se* (Figs 3 and 4). Instead, it reduces the advantage of having more sperm, though releasing more sperm is still weakly associated with higher paternity (Fig. 1, Table 1).

## Discussion

Our results show that females—through their ovarian fluid —can influence the nature and outcome of sperm competition among conspecific males, even in a species with external fertilization such as the ocellated wrasse (*Symphodus ocellatus*). Our results provide evidence that female ovarian fluid changes the dynamics of fertilization at the gametic level and suggest a mechanism by which this might occur. The presence of female ovarian fluid changed the relationship between sperm number and paternity in a way that decreases the fitness advantage of greater sperm production, and thereby increasing the relative importance of greater sperm velocity (Fig. 1, Table 1). Nesting males had greater sperm velocity and motility in general (Fig. 2), sperm characteristics known to be important for fertilization in a variety of species with external fertilization^46^,^47^,^48^. Nesting males thus release fewer^37^ but higher quality (Fig. 2) sperm. ovarian fluid further enhances sperm velocity, motility, straightness and chemoattraction for both sneaker and nesting males, which causes a shift from sperm number to sperm velocity driving the ‘race' to find the micropyle and fertilize the egg (Figs 3 and 4). Taken together, these mechanisms allow directional cryptic female choice among conspecific males to occur at the gametic level that likely exerts selection on male sperm allocation and characteristics through their effect on paternity. The differences between the male types in sperm characteristics and the effect of ovarian fluid on male sperm characteristics are likely the result of male adaptation to selection arising from the environment provided by the female's ovarian fluid during sperm competition. Such directional cryptic female choice could generate an evolutionary chase between female ovarian fluid and male sperm characteristics that would only be detected by detailed study of the mechanisms underlying fertilization^49^,^50^,^51^.

Under natural conditions, nesting males release fewer sperm than sneaker males (sneakers release four million sperm per spawn, while nesting males release only one million per spawn on average^37^). This female-driven effect on fertilization and greater nesting male sperm velocity may help explain how nesting males father on average two-third of the young in their nest^41^, despite being consistently at a disadvantage in terms of sperm number and experiencing a high risk and intensity of sperm competition from sneaker males^37^. While we found no direct effect of ovarian fluid on the relative success of alternative male phenotypes, this mechanism of cryptic female choice would under natural conditions in this species reduce the one advantage that sneakers otherwise have during sperm competition.

This effect of ovarian fluid on sperm competition also provides a mechanism by which a female trait could favour nesting male paternity—relative to what it would have been in the absence of this trait—during sperm competition between nesting and sneaker males. We know that parental males under natural conditions find themselves on the left side of the graph shown in Fig. 1 (because they release less sperm), while sneaker males find themselves on the right side of this graph (because they release more sperm). This means that the presence of ovarian fluid increases the paternity of the less common sperm (which under natural conditions is the nesting male's) relative to what it would have been in the absence of ovarian fluid (that is, on the left the predicted paternity of the male releasing fewer sperm is higher in the presence than in the absence of ovarian fluid). Given that this female-driven fertilization bias decreases the sperm number advantage that sneakers would otherwise have (and thereby increasing the relative importance of sperm velocity), this mechanism has the potential to alter selection not only on sperm allocation and characteristics, but also drive sexual selection among alternative male reproductive types because ovarian fluid favours the paternity of parental males relative to what it would be in the absence of female ovarian fluid.

In contrast to the results reported here, a recent study on Chinook salmon found no evidence of directional post-mating female choice among males^13^. This is somewhat surprising given that a female-driven effect of ovarian fluid on male sperm characteristics had previously been documented in this species^15^,^29^, and the potential fitness benefits of cryptic female choice in this species^21^. Study design could explain this difference in results. These studies^13^,^15^,^29^ examined whether the presence of female ovarian fluid drove female choice for genetic complementarity (that is, non-directional cryptic female choice^32^). By comparison, our study considered directional cryptic female choice between two discrete male phenotypes. Furthermore, selection for post-mating female choice between nesting and sneaker males is also likely to be strong in the ocellated wrasse. Female wrasses exhibit a strong pre-mating preference for nesting males^38^,^42^ but cannot avoid spawning with sneaker males because fertilization is external. A female preference for nesting males may have evolved because nesting males are both older and faster growing than sneaker males in this species^35^. Sneakers and nesting males represent two distinct life history trajectories, though the heritability of the pathways is not known^35^. Nonetheless, female traits that increase the fitness of one alternative reproductive male phenotype over another will exert sexual selection on the expression of these alternative male types even if condition-dependent in origin^52^,^53^. Female wrasses may also prefer nesting males because female fitness depends on male parental care, and higher siring success could select for greater levels of paternal care by the nesting males, if males respond directly to paternity^41^. Strong selection on females to have their offspring sired by nesting males could therefore explain why mechanisms have evolved in this species that could allow directional cryptic female choice by increasing the paternity of parental nesting males at the costs of non-parental sneaker males in the ocellated wrasse.

This study shows that female choice after mating can bias paternity toward preferred males—and therefore drive sexual selection on conspecific male phenotypes—even when fertilization is happening outside of the body of the female. These results also suggest the potential for sexually antagonistic selection arising from sexual conflict between females and sneaker males over fertilization that drive the co-evolution of not only male and female gametic traits, but could also result in co-evolution between female ovarian fluid and non-gametic components of the male phenotype (such as alternative male types and paternal care)^49^,^50^. It remains to be seen whether post-mating female choice that drives directional sexual selection on males will be common enough to play a general role in shaping the diversity of male traits in species with external fertilization. Here, we also find that the mechanism of cryptic female choice is relatively simple. Rather than a complex mechanism that favours a specific aspect of nesting male sperm, ovarian fluid simply reduces the advantage of releasing more sperm, and therefore likely favours the paternity of the preferred parental nesting male under natural conditions indirectly. In general, these results show that females can choose among males even after mating when fertilization is happening outside the female's body.

## Methods

### Study species and site

Research was performed at the University of Liege Marine Station (STARESO) and in the Baie de Revellata in the Mediterranean Sea (near Calvi, Corsica) using SCUBA. During the breeding season (May–June), mating occurs throughout the day^33^. Fertilization is external and sperm competition high^36^,^37^,^38^. Females choose among nests, but do not remain on a nesting male's territory or provide parental care. Conflict over mating exists between females and sneaker males, as females prefer to mate with nesting males, but cannot fully control parentage due to external fertilization. One-third of nesting males desert their nests without providing care, and nesting males that sire more offspring are more likely to provide care^41^. All aspects of this research were consistent with the legal requirements of the country in which the work was carried out, covered by animal care protocols approved by the Institutional Animal Care and Use Committees of Yale University and the University of California Santa Cruz, and conform to the American Society for the AB/ABS Guidelines for the Use of Animals in Research. Females, sneakers and nesting males used in this study were all collected from actively spawning nests from the Baie de Revellata in the Mediterranean Sea (near Calvi, Corsica). Fish were not reused either within or between experiments.

### The effect of ovarian fluid on sperm competition

To remove behavioural effects on paternity, we first performed *in vitro* sperm competition trials (*n*=30, 10 per treatment). In this first experiment, eggs were stripped from a female into a Petri dish. The ovarian fluid was either (1) left untouched (ovarian fluid baseline), (2) removed with a 10 μl micropipette and then the eggs rinsed with fresh seawater (ovarian fluid removed) or (3) removed but added back to the eggs after rinsing as a manipulation control (ovarian fluid replaced). We collected 2 μl of milt from one sneaker and one nesting male per replicate and then simultaneously activated each sperm sample in 1 ml seawater and added 200 μl of the activated sperm solution from both the sneaker and nesting male simultaneously to the eggs. Each replicate was done on a different set of sneaker and nesting males, and males were given an individual mark before release to avoid accidental recapture and reuse within or between experiments. A 2 μl subsample of the activated sperm of both males was then added to two different chambers of a 20 μm, four-chamber Leja slide and videotaped under × 10 negative-phase contrast using a Motic BA310 light microscope and 60 Hz EIA monochrome RS-170 camera for later analysis of sperm characteristics. We determined sperm velocity, motility, linearity and straightness for each male, using a Hamilton Thorne CEROS CASA system. The remaining sperm samples were preserved in 3% formalin and the number of sperm in a subsample counted (at × 400 bright-field illumination) later to determine the concentration of sperm in the sample.

The fertilized eggs were covered with seawater, left for 5 min to ensure fertilization, rinsed with fresh seawater and then held in aquaria with fresh flowing seawater in 100 μm Nitex plankton netting. The eggs were allowed to develop for 3–5 days (until pigmented eye development) and then preserved in 80% ethanol for paternity analyses. Small (2 mm^2^) fin clips were used to genotype adults. Although a few eggs showed no sign of early development (based on the absence of cell division), consistent with a failure to be fertilized (perhaps due to egg inviability), most eggs that showed signs of fertilization developed normally and were collected for paternity analyses.

Parentage was assigned to either the nesting or sneaker male using microsatellite-based genetic paternity assignment. DNA from females, nesting males and sneakers was extracted using a Qiagen DNAeasy Blood and Tissue Kit, while DNA from eggs extracted using a magnetic-bead-mediated robotic extraction, performed using Verde Labs Genomic DNA Extraction Chemistry on a Biosprint96 Extraction Robot. Samples were amplified using six microsatellite loci developed for *S. ocellatus*^54^ (Soc1017, Soc1063, Soc1109, Soc1198, Soc3121 and Soc3200, Primer sequences are given in the Supplementary Table 1) and previously used for paternity assignment^41^. Primer sets were used in combination in a PCR reaction (multiplexing^55^), and amplified using a DNA Engine Tetrad 2 Thermal Cycler from Biorad, set at the following parameters for both triplex reactions: 94 °C for 120 s; 15 cycles of 94 °C for 30 s, 60–54 °C for 30 s (starting at 60 °C on first cycle and decreasing by 0.5 °C for each subsequent cycle), 72 °C for 90 s; 23 cycles of 94 °C for 30 s, 54 °C for 30 s, 72 °C for 90 s; ending 72 °C for 600 s (10 min). PCR product was run for fragment analysis on an Applied Biosystem 3,730 DNA Analyzer, visualized and scored using the standard protocol for Genemarker software from Softgenetics using a bin-set not previously made. Peaks were evaluated and scored visually by an observer blind to sample identity.

The number of alleles per locus was 17–37 (mean=25.0), and expected heterozygosity ranged from 0.73 to 0.90 (mean=0.87). Combined non-exclusion probability for the first parent is 0.00185. Nesting and sneaker males were assigned as the father based on strict exclusion (if a male did not share an allele at each locus that could be compared, he was excluded as the father). This method has previously been compared with assignment allowing one mis-match, and assignment rates do not substantially differ between methods^41^. Maternal genotype was used to assign a father when both males were potential matches, and offspring for whom neither male could be excluded even when consideration of maternal genotype were excluded from the analysis.

### The effect of ovarian fluid on sperm characteristics

In a second experiment, we compared the effect of ovarian fluid on sperm characteristics. Sneaker and nesting males were observed, caught and their gametes collected as described above (*n*=16 sneaker males and *n*=16 nesting males, males were not used more than once either within or between experiments). For this study, we collected and activated 3 μl of milt from a single male in 0.5 ml of fresh seawater. In all, 2 μl of the activated sperm were then mixed with 4 μl of fresh seawater or 2 μl seawater and 2 μl of ovarian fluid from a single female (yielding two samples—either with or without ovarian fluid —from each male with the same concentration of milt). Two 2 μl samples of activated sperm from the same male (one with and one without ovarian fluid from 16 sneaker males and 16 nesting males) were then loaded into two adjacent chambers of a 20 μm, four-chamber Leja slide, videotaped simultaneously and analysed using a Hamilton Thorne CEROS CASA system as described above. Data collection and analysis were therefore paired within individual male (that is, the data on sperm characteristics in the presence and absence of ovarian fluid were paired for an individual male).

### The effect of ovarian fluid on sperm chemoattraction

In a third experiment, we examined the effect of eggs and ovarian fluid on sperm chemoattraction using a cell migration assay modified from earlier work on salmon^8^ using Corning Transwell Polycarbonate Membranes with 8 μm diameter pores at a density of 1 × 10^5^ pores cm^−2^. Sneaker and nesting males were observed, caught and their gametes collected as described above. We compared the number of sperm to cross the membrane with and without ovarian fluid for sperm collected from either a sneaker or a nesting male (*n*=16 sperm from sneaker males with eggs and ovarian fluid, *n*=15 sperm from a sneaker male without ovarian fluid, *n*=15 with sperm from a nesting male with ovarian fluid and *n*=13 sperm from a nesting male with only seawater). Males were not used more than once either within or between experiments. For the ovarian fluid treatment, eggs and ovarian fluid were stripped from a female into the bottom cell of a Transwell plate. We added 1 ml seawater to first the bottom and then the top chamber of the Transwell. We then collected 3 μl milt from either a sneaker or nesting male and activated the sperm in the seawater of the upper chamber. After 5 min, we collected the samples from the top and bottom of the Transwell, noting their volume. These samples were preserved in 3% formalin, stained with Rose Bengal and their concentration determined later using DRM-600 CELL-VU Sperm Counting Chambers (disposable cytometers). These data allowed us to estimate the number of sperm originally introduced above the membrane as well as the number that passed through the wrasse egg micropyle-sized pores, and whether it depended on male type or the presence/absence of ovarian fluid and eggs.

### Statistical analyses

All analyses were done in R (version 3.2). To determine the effect of the presence or absence of ovarian fluid on sperm competition and nesting male paternity (the first experiment), we ran general linear logistic regression models (assuming a quasibinomial distribution of the errors due to overdispersion of the data, see Table 1 for full model structure and results)^56^. We also performed hierarchical model comparison and found that the best-fit model based on comparison of the AIC (using anova and AICctab from library bbmle in R). To compare the sperm characteristics of the nesting and sneaker male pairs used in the *in vitro* fertilization trials, we used between-male repeated measures mixed effects models fit by maximum likelihood (see Table 2 for full model structure and results). To examine the effect of ovarian fluid on male sperm characteristics (the second experiment), we used within male (with and without ovarian fluid) repeated measures mixed effect models fit by maximum likelihood (see Fig. 3 for full model details and results). To examine the effect of ovarian fluid on sperm chemoattraction (the third experiment), we used a two-way ANOVA (male type × ovarian fluid treatment, see Fig. 4 for full model details and results) on the number of sperm that migrated to the bottom chamber as well as a general linear logistic regression to examine the probability sperm would cross the membrane to the eggs (also see Fig. 4 for full model details and results)^56^.

### Data availability

Data associated with this article (that is from the three experiments) are available from the Dryad Digital Repository: http://dx.doi.org/10.5061/dryad.1m056 (ref. 58). For the first experiment, the data are information on ovarian fluid treatment, the number of offspring genotyped, the number of offspring assigned to the nesting male, the estimated concentration of sperm in the sample collected from the sneaker and nesting male involved in each cross and paired data on the sperm characteristics for both males used in each *in vitro* cross. The data from the second experiment include male type (sneaker or nesting male), ovarian fluid treatment (present or absent) and sperm characteristics for each replicate. The data for the third experiment are male type, ovarian fluid treatment and the estimated number of sperm above and below the membrane.

## Additional information

**How to cite this article:** Alonzo, S. H. *et al.* Ovarian fluid allows directional cryptic female choice despite external fertilization. *Nat. Commun.* 7:12452 doi: 10.1038/ncomms12452 (2016).

## Supplementary Material

Supplementary Information Supplementary Table 1 and Supplementary References

## Figures and Tables

**Table 1:** Ovarian fluid changes the relationship between sperm number and paternity.

Variable	Parameter estimate	s.e.m.	*t* value	P(>|t|)
**Intercept**	−1.834	0.5569	−3.293	**0.003**
Effect of treatment: OF replaced compared with OF baseline	1.257	0.6555	1.917	0.068
Effect of treatment: OF rinsed compared with OF baseline	0.09538	0.6316	0.151	0.881
**Effect of relative sperm number (nesting male: sneaker)**	0.00028	0.0001	2.887	**0.009**
**Effect of relative sperm velocity (VCL, nesting male: sneaker)**	0.00005	0.00002	2.690	**0.013**
Interaction between: effect of OF replaced (relative to OF baseline), and relative sperm number	−0.00019	0.00018	−1.066	0.298
**Interaction between: effect of OF rinsed (relative to OF baseline), and relative sperm number**	−0.00029	0.00013	−2.608	**0.016**

OF, ovarian fluid.

Results of the best-fit general linear logistic regression model predicting the probability of paternity as a function of ovarian fluid treatment, sperm number and velocity. Significant effects are highlighted as bold font. GLM Model: Pr(nesting male paternity, sneaker male paternity)=Relative sperm number(NM:SN) × Treatment+Relative sperm velocity (VCL NM:SN) (using glm family=quasibinomial in R). Dispersion parameter for quasibinomial family taken to be 2.437. Null deviance: 115.246 on 28 degrees of freedom. Residual deviance: 58.677 on 22 degrees of freedom. *N*=30 *in vitro* crosses (10 per treatment). Each cross involved a female, sneaker and nesting male, and fish were not used more than once either within or between experiments.

**Table 2:** Nesting males and sneakers differ in sperm motility, velocity and longevity.

Variable		Sperm motility (% motile)		Curvilinear sperm velocity (VCL)	
	df	*t* value	P (>|t|)	*t* value	P (>|t|)
**Intercept**	87	13.93	**<0.001**	16.74	**<0.001**
**Effect of male type: sneaker relative to nesting male**	87	−3.96	**<0.001**	−1.80	0.076
**Effect of time: 50 s relative to 40 s**	58	−2.25	**0.028**	−4.93	**<0.001**
**Effect of time: 60 s relative to 40 s**	58	−4.91	**<0.001**	−7.84	**<0.001**
Interaction between: the effect of male type (sneaker relative to nesting male), and the effect of time (50 s relative to 40 s)	87	1.19	0.238	1.82	0.071
**Interaction between: the effect of male type (sneaker relative to nesting male), and the effect of time (60 s relative to 40 s)**	87	2.23	**0.028**	2.749	**0.007**

Results are shown for sperm motility and curvilinear velocity. Other measures of velocity yield qualitatively similar results. No differences in straightness or linearity were detected. The *P* values of significant effects are highlighted as bold font. A linear mixed effects model was fit by maximum likelihood with time (40, 50 and 60 s) as a repeated measure and cross as a random effect (using lme from library nlme in R version 3.2). Fixed effects were: motility∼Type × Repeat; VCL∼Type × Repeat (Repeat=40, 50, 60 s). *n*=30 *in vitro* crosses (10 per treatment). Each cross involved a new female, sneaker and nesting male. Fish were not used more than once either within or between experiments.
