# Supplementary material for: Ovarian fluid allows directional cryptic female choice despite external fertilization
Source: Nat Commun. 2016 Aug 16;7:12452. doi: 10.1038/ncomms12452 (PMC4990696; doi:10.1038/ncomms12452)
Supplement: Supplementary Information — Supplementary Table 1 and Supplementary References [file ncomms12452-s1.pdf]

Supplementary Tables

Supplementary Table 1

Characterization of the six [sic] *Symphodus ocellatus* microsatellite loci used in this study (based on five populations and 10 individuals per population, this information is based on a partial reprint of Table 1 from Arigoni & Largiader 2000[1] for the six loci used in this study).

Locus	Repeat Array	Primer sequences (5' → 3')	Annealing temp (°C)	$MgCl_2$ (mM)	Number of alleles	Size range (bp)**	H_O	H_E
Soc1017PBBE	$(AC)_{20}GC(AC)_2$	*TCC TGT CAG TCT CCC TTC A<br>GTG ATT GAT TAG GCG ATG AG	63	0.8	20<br>(9-15)	77-123<br>(101)	0.86<br>(0.6-1.0)	0.92<br>(0.87-0.96)
Soc1063PBBE	$(GA)_2(GT)_8AT(GT)_4$	*CCC TTC TTG TGT CAT TCC AAG<br>CCT CAC TTG ATA TGT CC	56	0.8	14<br>(6-9)	92-134<br>(98)	0.76<br>(0.4-1.0)	0.86<br>(0.83-0.91)
Soc1109PBBE	$(GT)_{10}$	*AGG ATT TAG CCT GCC CAG GA<br>TGC GGT GAA TGG CTG TAG GT	57	1.0	13<br>(7-9)	133-167<br>(137)	0.84<br>(0.7-1.0)	0.87<br>(0.80-0.89)
Soc1198PBBE	$(TG)_5TA(TG)_{13}$	*CTC TTT CTG CCT GCA CTC GAC<br>TTC ATT GGA CAG CAC AC	57	1.2	11<br>(5-9)	89-113<br>(109)	0.76<br>(0.6-0.9)	0.80<br>(0.67-0.89)
Soc3121PBBE	$(GT)_{18}$	*ACG ACA AGC TGC ACG AAC<br>CCA GTA ATT CTG ACT CCA CCC	56	0.9	28<br>(12-14)	82-205<br>(102)	0.90<br>(0.8-1.0)	0.95<br>(0.94-0.96)
Soc3200PBBE	$(GT)_{15}$	*AGT GCC AGA TGT ATA TGG G<br>CAT GGA CGC ATT TGT AGC	51	1.0	27<br>(12-14)	120-188<br>(134)	0.88<br>(0.8-1.0)	0.94<br>(0.91-0.96)

The sequences of cloned fragments have GenBank accession nos AJ278566–AJ278572; *HO,* observed heterozygosity; *HE,* expected heterozygosity; given are the mean values across the five populations with range in parentheses. *Primer used for end-labelling. **Cloned insert size in parentheses.

Supplementary References

1. Arigoni, S. & Largiadèr, C. R. Isolation and characterization of microsatellite loci from the ocellated wrasse *Symphodus ocellatus* (Perciformes: Labridae) and their applicability to related taxa. *Molecular Ecology* **9**, 2166-2169, (2000).
